# Gut–Liver Immune Response and Gut Microbiota Profiling Reveal the Pathogenic Mechanisms of *Vibrio harveyi* in Pearl Gentian Grouper (*Epinephelus lanceolatus* **♂** × *E. fuscoguttatus* **♀**)

**DOI:** 10.3389/fimmu.2020.607754

**Published:** 2020-11-26

**Authors:** Yiqin Deng, Yaqiu Zhang, Haoxiang Chen, Liwen Xu, Qian Wang, Juan Feng

**Affiliations:** ^1^ Key Laboratory of South China Sea Fishery Resources Exploitation and Utilization, Ministry of Agriculture and Rural Affairs, South China Sea Fisheries Research Institute, Chinese Academy of Fishery Sciences, Guangzhou, China; ^2^ Tropical Aquaculture Research and Development Centre, South China Sea Fisheries Research Institute, Chinese Academy of Fishery Sciences, Hainan, China; ^3^ College of Fisheries and Life Science, Shanghai Ocean University, Shanghai, China

**Keywords:** *Vibrio harveyi*, pearl gentian grouper, immune response, gut microbiota, interplay, pathogenesis

## Abstract

*Vibrio harveyi* causes vibriosis in nearly 70% of grouper (*Epinephelus* sp.), seriously limiting grouper culture. As well as directly inhibiting pathogens, the gut microbiota plays critical roles in immune homeostasis and provides essential health benefits to its host. However, there is still little information about the variations in the immune response to *V. harveyi* infection and the gut microbiota of grouper. To understand the virulence mechanism of *V. harveyi* in the pearl gentian grouper, we investigated the variations in the pathological changes, immune responses, and gut bacterial communities of pearl gentian grouper after exposure to differently virulent *V. harveyi* strains. Obvious histopathological changes were detected in heart, kidney, and liver. In particular, nodules appeared and huge numbers of *V. harveyi* cells colonized the liver at 12 h postinfection (hpi) with highly virulent *V. harveyi.* Although no *V. harveyi* was detected in the gut, the infection simultaneously induced a gut-liver immune response. In particular, the expression of 8 genes associated with cellular immune processes, including genes encoding inflammatory cytokines and receptors, and pattern recognition proteins, was markedly induced by *V. harveyi* infection, especially with the highly virulent *V. harveyi* strain. *V. harveyi* infection also induced significant changes in gut bacterial community, in which *Vibrio* and *Photobacterium* increased but *Bradyrhizobium*, *Lactobacillus*, *Blautia*, and *Faecalibaculum* decreased in the group infected with the highly virulent strain, with accounting for 82.01% dissimilarity. Correspondingly, four bacterial functions related to bacterial pathogenesis were increased by infection with highly virulent *V. harveyi*, whereas functions involving metabolism and genetic information processing were reduced. These findings indicate that *V. harveyi* colonizes the liver and induces a gut-liver immune response that substantially disrupts the composition of and interspecies interactions in the bacterial community in fish gut, thereby altering the gut-microbiota-mediated functions and inducing fish death.

## Introduction

*Vibrio harveyi* is an opportunistic pathogen that causes fatal vibriosis in aquatic vertebrates and invertebrates, leading to significant morbidity and mortality among a diverse range of fish species worldwide (1–3). It is reported that *V. harveyi* causes vibriosis in nearly 70% of grouper (*Epinephelus* sp.), a commercially important fisheries species in China, with a yield of 183,127 tonnnes in 2019 (4). *V. harveyi* usually infects its host *via* the processes of adhesion, invasion, reproduction, and toxin release, ultimately leading to the host’s death (5, 6). Although several virulence determinants have been identified in *V. harveyi*, including extracellular products, lipopolysaccharide, bacteriophages, and quorum-sensing factors, empirical evidence of the host-pathogenic process in response to infection is otherwise sparse.

The most frequent clinical signs of *V. harveyi*-infected fish include lethargy, anorexia, erratic swimming, deep dermal lesions, skin ulcer, and eye lesions (7–9). The visceral organs of affected fish become congested and inflamed, particularly the liver, spleen, and kidney, resulting into gastroenteritis, vasculitis, septicemia, etc. (10–12). Fish have many nonspecific and specific immune mechanisms to eliminate pathogens during their attachment, penetration, survival, and spread within the host, thus resisting bacterial diseases (13). The gut-liver immune system has gradually become recognized in mammals, and its dysregulation is associated with many gut and liver diseases (14). The gut-associated lymphoid tissue (GALT) is an important constituent of the mucosal immune system, and constitutes a local immune environment of both defensive and tolerance (15). The liver is a central immunological organ, and is continuously exposed circulating antigens and endotoxins from the gut microbiota (16). Because it is adjacent to the GALT, it contributes to immune surveillance (14). In fish, the gut-liver immunity of tilapias infected with *Streptococcus agalactiae* has been comprehensively investigated by Wu et al. (17), with integrated transcriptomic and proteomic studies. Those data suggest that the fish gut and liver collaborate immunologically, maintaining immunological homeostasis with specific strategies. However, the fish gut-liver immune system remains largely unknown, including in the grouper after *V. harveyi* infection, although both enteritis and liver-and-gallbladder syndrome frequently occur, and have become limitations to grouper aquaculture (18, 19).

More than one billion microorganisms, predominantly bacteria, colonize the gastrointestinal tract and establish a mutualistic relationship with fish (20). However, when the mutualistic relationship between the host and its microbiota is disrupted, the gut microbiota can cause or contribute to disease (21, 22), including vibriosis, furunculosis, enteric septicemia, and aeromoniasis in fish (23, 24). For example, the relative abundances of Proteobacteria, Fusobacteria, Bacteroidetes, and Firmicutes change in *Ctenopharyngodon idellus* after *Aeromonas hydrophila* challenge, leading to the deformation of the intestinal villi, redness and congestion at the injection site, and even the death of the fish (25). In addition to digesting and fermenting carbohydrates and producing vitamins, the gut microbiota plays critical roles in the development of the GALT, the activation of the gut immune responses, and the prevention of colonization by pathogens, thus resisting disease and maintaining the homeostasis of the gut (20, 26). For example, infection with *Salmonella* in mice induces the processing of pro-interleukin 1β (pro-IL1β) (27). The consumption of *Bifidobacterium* in mice increased the number of regulatory T cells, and attenuated the severity of intestinal disease after *Salmonella* infection (28). In turn, the gut immune responses that are induced by commensal populations regulate the composition of the microbiota (29). Therefore, the changes in the gut microbiota caused by *V. harveyi* infection play an important role in fish resistance to *V. harveyi* infection and warrant further study.

In the present study, the pearl gentian grouper (*Epinephelus lanceolatus* ♂ × *E. fuscoguttatus* ♀) was selected as the research object. A histopathological analysis, the expression of immune-related genes, and the structure and function of the intestinal microbial community of the pearl gentian grouper were investigated after infection with a high- or low-virulence strain of *V. harveyi*. This design allowed us: (i) to assess how the interspecies interactions and functions of the gut bacteria were altered during the exacerbation of disease in the grouper; (ii) to screen sensitive gut taxa, e.g., bioindicators that are closely associated with the progression of *V. harveyi* infection; (iii) to evaluate the associations among *V. harveyi* infection, the gut microbiota, and the immune responses of the grouper; and (iv) to provide a scientific basis for immunological or microbe-based therapies for *V. harveyi* disease in the pearl gentian grouper.

## Materials and Methods

### Bacterial Strains and Growth Conditions

*V. harveyi* 345:pMMB207 containing the chloramphenicol resistant plasmid pMMB207 (30) was originated from the *V. harveyi* 345 strain which was previously isolated from a diseased pearl gentian grouper by our group (31). The chloramphenicol resistant was used as the selection marker for bacterial pathogen load analysis. *V. harveyi* 345:pMMB207 was shown to have a median lethal dose (LD_50_) of 4.49 × 10^4^ CFU g^−1^ in pearl gentian (data not published). An *hfq* deletion mutant was constructed with two rounds of allelic exchange (32) (data not published). When a grouper was injected with a 100 µL of culture containing 7.5LD_50_ (a final dosage of 3.37 × 10^5^ CFU g^−1^) wild-type strain *V. harveyi* 345:pMMB207, the mortality rate was 41.11% at 12 h postinjection (hpi), 83.33% at 24 hpi, 86.70% at 48 hpi, and 86.70% at 108 hpi, whereas when a grouper was injected with the same amount the *hfq*-deletion mutant strain *V. harveyi* 345Δ*hfq*:pMMB207, the cumulative mortality rate was 0.00% at 108 hpi (**Figure S1**). Therefore, we defined these two strains as high-virulence and low-virulence *V. harveyi*, respectively. They were cultured in Luria–Bertani (LB) broth with 2% additional NaCl (LBS) containing 34 µg mL^−1^ chloramphenicol (Cm) at 28°C.

### Fish Infection

The pearl gentian groupers used for the infection assay were purchased from a local aquaculture farm in Shenzhen, China, and had a mean weight of 50 ± 2 g. The groupers were cultured in aerated recycled seawater at room temperature and fed twice daily (8:00 am and 4:00 pm) with No. C5 commercial feed (Guangdong, the group of Marine biological research and development co., LTD). After acclimation for 2 weeks, 135 vigorous fish were randomly divided into three groups (groups A, B, and C; 15 fish per tank in triplicate for each group) and stop feeding before 24 h of injection. The high-virulence (group A) and low-virulence (group B) *V. harveyi* strains were cultured on glass slants overnight and resuspended in 3 mL normal saline. The cells were diluted to an optical density at a wavelength of 600 nm (OD_600_) of 1.85 with normal saline. The fish in groups A and B were injected intraperitoneally (i.p.) with 100 μL of the diluted high-virulence and low-virulence *V. harveyi* cultures (7.5LD_50_ and a final dosage of 3.37 × 10^5^ CFU g^−1^), respectively, and the control fish (group C) were injected i.p. with 100 μL of normal saline. And there were no feedings after the injection.

### Sampling

For each time point, one fish was randomly selected from each tank and totally three fishes were selected for each group (including group A, group B, and group C) and, sampled and dissected at 12, 24, and 48 hpi. Liver samples (30–50 mg) were collected on ice at 12, 24, and 48 hpi for a pathogen load analysis. The brain, heart, liver, spleen, gills, kidneys, and gut of each fish were fixed in 10% buffered formalin at 12 hpi for histopathological examination. Liver and gut samples (50–100 mg each) were soaked in 1 mL of RNAstore Reagent (Tiangen, Beijing, China) at 12 hpi, before RNA extraction and quantitative PCR. The whole gut contents were collected in sterile tubes at 12 hpi and immediately stored at −80°C before DNA extraction and gut microbiotal analysis.

### Bacterial Pathogen Load Assay

The pathogen load of the liver by the two *V. harveyi* strains was determined based on the study of Whitaker et al. (33), with some modification. The livers were homogenized in 500 μL of normal saline and the homogenized samples were 10-fold serially diluted with normal saline, and coated onto LBS medium containing 34 μg/mL Cm. The plates were incubated at 28 °C overnight. The numbers of bacterial colonies were recorded for the pathogen load analysis.

### Histopathological Analysis

Samples were fixed in 10% buffered formalin for at least 24 h. The tissues were then dehydrated, embedded in paraffin, and cut into sections (4 μm thick) with a rotary microtome RM2135 (Leica, Wetzlar, Germany). The sections were stained with hematoxylin and eosin (HE) with standard techniques for histopathological evaluation. All sections were observed under an optical Leica DFC495microscope (Ernst Leitz, Wetzlar, Germany).

### Total RNA Extraction, cDNA Synthesis, and Gene Expression

All reagents were from Takara (Takara Bio Inc., Shiga, Japan). According to the instructions, total RNA was extracted from the liver and gut with RNAiso Plus. Reverse transcription (RT) was performed with PrimeScript™ RT Reagent Kit with gDNA Eraser. Quantitative PCR was used to analyze gene expression with TB Green™ Premix Ex Taq™ Π (Tli RnaseH Plus). Relative expression was calculated with the 2^−ΔΔCT^ method (34), using the gene β-actin as the endogenous control, and was normalized to the values for the gut samples in control group C. The primers used in this study are listed in **Table S1**.

### Gut Microbiotal Composition and Function

The total genomic DNA of the fish gut contents was extracted with the cetyl trimethylammonium bromide (CTAB) method. A 16S rRNA library was generated, sequenced, and analyzed as previously described (35). The functional capacity of each microbiota was predicted from the 16S rRNA gene sequence data with the Tax4Fun software (36). The 16S rRNA sequences were screened against the SILVA database with a BLAST search to obtain the functional annotation information. The functional pathways were annotated with the Kyoto Encyclopedia of Genes and Genomes (KEGG) orthology groups (37) at levels 1, 2, and 3.

### Statistical Analysis

A Bray–Curtis distance matrix was constructed for the bacterial community and function analyses with preliminary one-way permutational multivariate analysis of variance (PERMANOVA) and a principal co-ordinates analysis (PCoA). PERMANOVA was used to examine the diversity of the bacterial communities and functions in the different groups (*p* < 0.05 was considered statistically significant). PCoA was used to investigate the succession of microbiotal assemblages through the different groups. A SIMPER analysis was used to identify the bacterial taxa driving the differences among the different groups. One-way ANOVA was used to examine the variations in gene expression and α-diversity. A *t* test was used to examine the variations in pathogen load and bacterial functions. Pearson’s correlation analysis was performed to determine the relationships among and within the gut bacterial communities, the predicted functions, and the expression levels of immunity-related genes. All statistical analyses were performed with PRIMER v6 and PERMANOVA+ (38) or IBM SPSS Statistics 19.0 (39). The pathogen load, expression levels of immune-related genes, and the relative abundances of phyla, genera, and bacterial function were shown as means ± SEM.

## Results

### Comparative Pathogen Load and Histopathology of Groupers Infected With High- or Low-Virulence *V. harveyi* Strains

**Figure 1:**
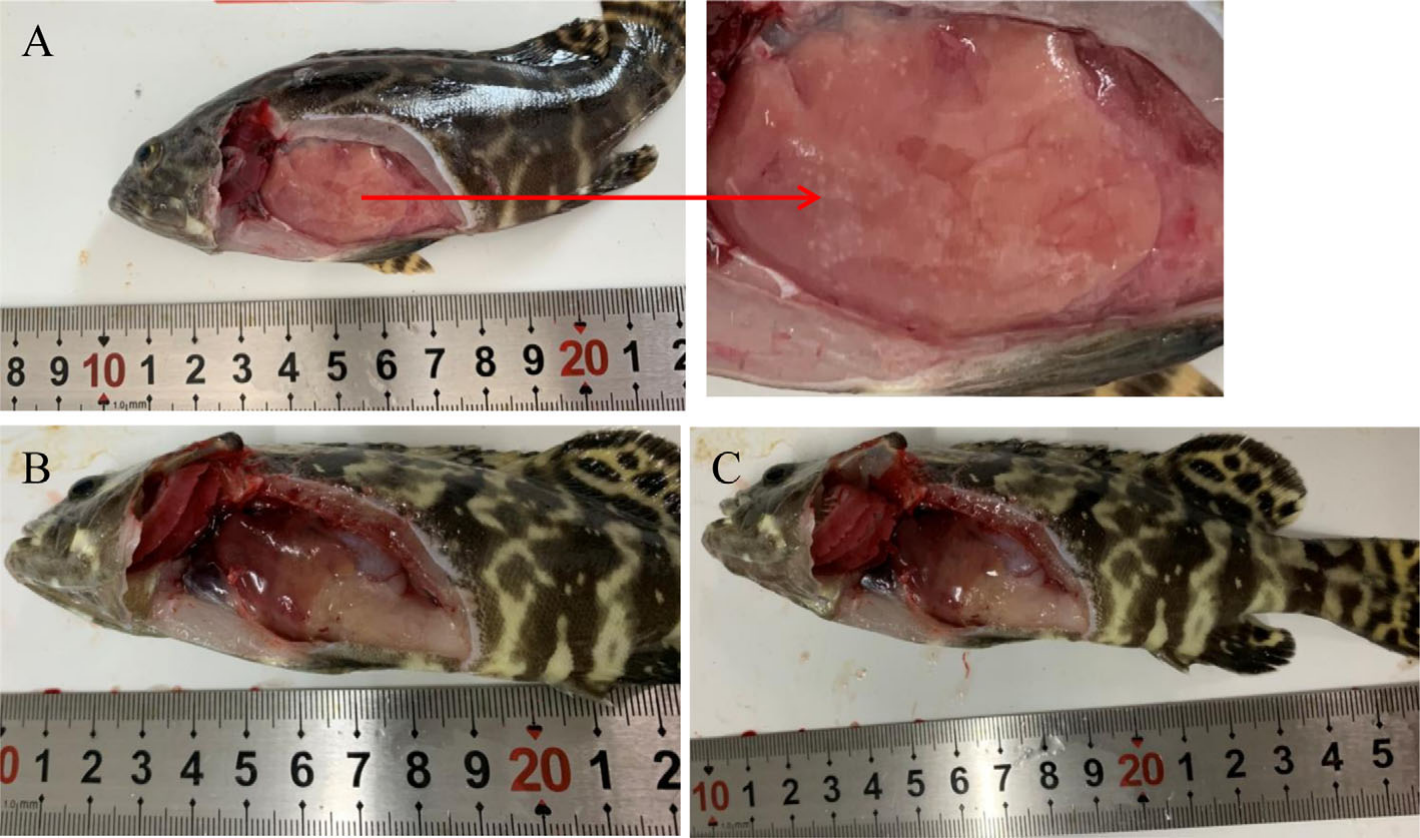
Pathological features of fish infected with high-virulence *V. harveyi* strain **(A)**, low-virulence *V. harveyi* strain **(B)**, or treated with normal saline **(C)**.

**Figure 2:**
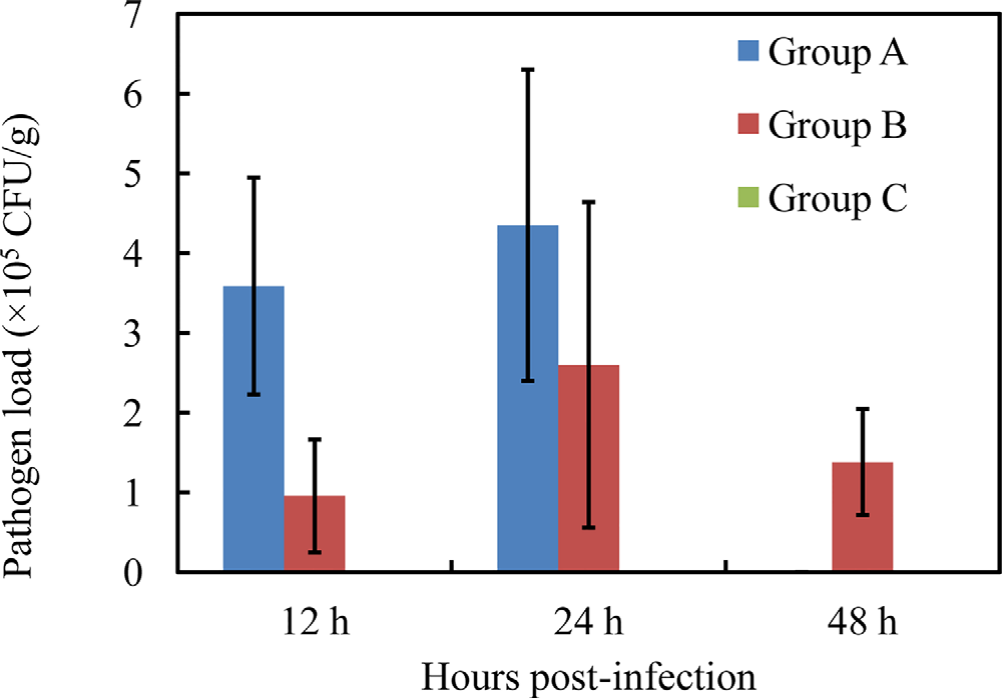
Liver pathogen load by high-virulence *V. harveyi* strain (Group A), low-virulence *V. harveyi* strain (Group B), and in normal-saline-treated fish (Group C). Values are means ± SEM (n = 3).

Nodules appeared in the liver at 12 hpi after injection with high-virulence *V. harveyi* (**Figure 1A**) but not with low-virulence *V. harveyi* or normal saline (**Figures 1B–C**). A pathogen load analysis indicated that the average bacterial pathogen load of the liver by high-virulence *V. harveyi* was 3.77- and 1.67-fold higher than that by low-virulence *V. harveyi* at 12 hpi and 24 hpi, respectively. Pathogen load with both *V. harveyi* strains increased at first and then decreased, reaching the highest value at 24 hpi. No *V. harveyi* was detected in the normal-saline-treated control group (**Figure 2**).

**Figure 3:**
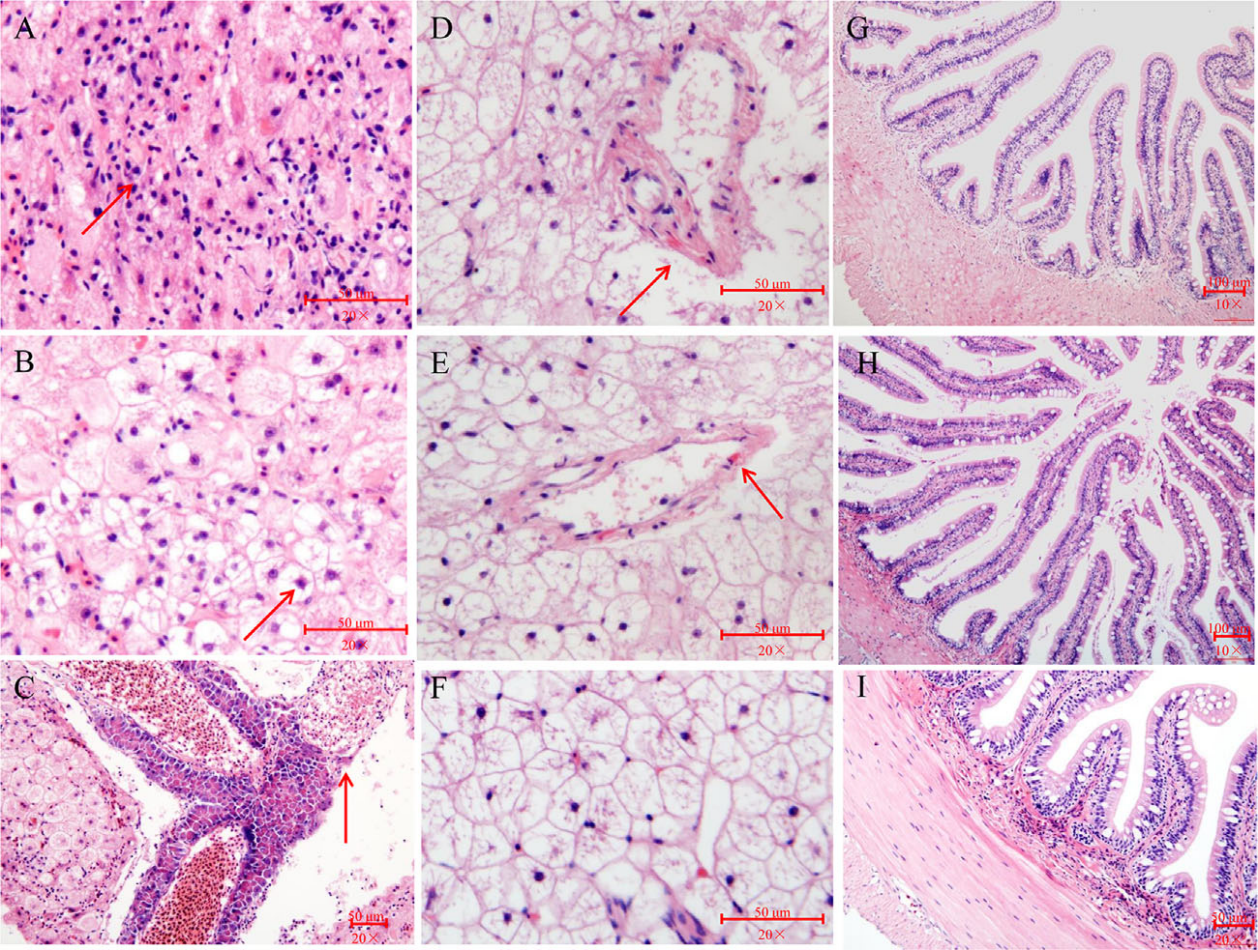
Histopathological changes in liver and gut of pearl gentian grouper after infection with *V. harveyi*. **(A–C)** Liver after infection with high-virulence *V. harveyi*. **(D** and **E)** Liver after infection with low-virulence *V. harveyi*. (**F**) Liver in fish treated with normal saline. **(G)** Gut after infection with high-virulence *V. harveyi*. **(H)** Gut after infection with low-virulence *V. harveyi*. **(I)** Gut in fish treated with normal saline. Arrows point to lesions.

No obvious histopathological changes was seen in the brain, spleen, gill, or gut at 12 hpi (**Figures S2A–C, G–I, J–L** and **Figures 3G–I**). The hearts of the *V. harveyi*-infected fish had moderate bacterial myocarditis and little parenchymal infiltration by inflammatory cells (**Figures S2D, E**). The kidneys of the *V. harveyi-*infected fish showed hemorrhage in the renal interstitial tissues, associated with the dissolution of the tubules (**Figures S2M, N**). The most serious histopathological changes were seen in the liver. Vacuolar degeneration and karyorrhectic debris were present in the livers of the group A fish, accompanied by the dissolution of the pancreatic epithelial cells (**Figures 3A–C**). Venous blood vessel loss and connective tissue necrosis were present in the livers of the group B fish (**Figures 3D, E**). No histopathological changes were detected in the heart, kidney, or liver in the control group (**Figures S2F, O** and **Figure 3F**), and the histopathological damage was more serious in group A than in group B (**Figures S2** and **S3**).

### Gut–Liver Immune Responses of Grouper to High-Virulence and Low-Virulence *V. harveyi* Strains

**Figure 4:**
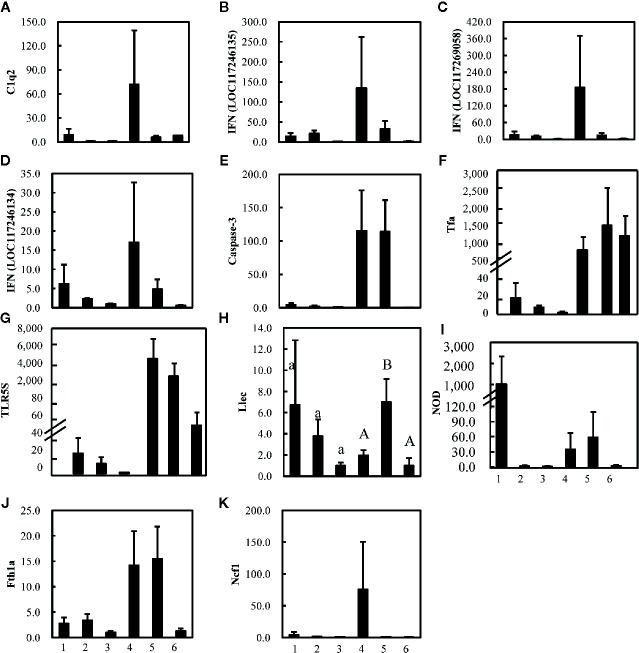
Expression levels of 11 **(A–K)** strongly altered immune-related genes in gut (1, 2, and 3) and liver (4, 5, and 6) of pearl gentian grouper after infection with high-virulence *V. harveyi* (1, 4), low-virulence *V. harveyi* (2, 5), or in fish treated with normal saline (3, 6). Expression was normalized to the expression in the gut of pearl gentian grouper treated with normal saline (3) and the ordinate is the normalized expression ratio. Except **Figure 4H** (Llec), no significantly differences were detected in other genes. Lowercase letters represent the results of one-way ANOVA of genes expression in gut (1, 2, and 3), and capital represent the results of one-way ANOVA of genes expression in liver (4, 5, and 6). Different letters **(A**, **B)** represent significant difference, and the same letters **(A)** represent no significant difference.

In the study of Wu et al. (17), 27 immune-related genes associated with nine different immune processes (**Table S1**) were selected to analyze the gut-liver immune response of the pearl gentian grouper to differently virulent *V. harveyi* strains. Gut advantage transcripts (the expression levels of gut and liver in control group were compared and the average expression altered more than 8-folds between these two control groups) encoded caspase (**Figure 4E**), the C-C motif receptor (CCR) (**Figure S3H**), C-reactive protein (CRR) (**Figure S3O**), and neuronal cell adhesion molecule (NCAM) (**Figure S3P**), whereas the liver advantage transcripts encoded C1q (**Figure 4A**), transferrin (**Figure 4F**), toll-like receptor (TLR) (Figure GL), and Kruppel-like factor (**Figure S3L**).

Although the expression of most genes were not significantly altered, the average expression of most genes varied strongly after injection with the *V. harveyi* strains (**Figure 4**). The gene encoding C1q (**Figure 4A**), which is involved in the immune process in the complement system, three genes encoding interferon (IFN)-induced proteins (**Figures 4B–D**), belonging to the immune process of inflammatory cytokines and receptors, two genes encoding caspase or transferrin and receptor (**Figures 4E, F**) and belonging to the immune process of other genes related to immune cell response, three genes encoding TLR, lectin, and nucleotide oligomerization domain (NOD) (**Figures 4G–I**), and belonging to the immune process of pattern recognition genes, one gene encoding ferritin (**Figure 4J**) and belonging to the immune process of acute phase reactions, and one gene encoding neutrophil cytosolic factor (NCF) (**Figure 4K**) belonging to the immune process of innate immune cells related, were upregulated in the gut and liver after injection with the high- or low-virulence *V. harveyi* strain. The expression of most of these genes tended to be higher after injection with the high-virulence *V. harveyi* strain than with the low-virulence *V. harveyi* strain.

### Distribution of Taxa in the Fish Gut

**Figure 5:**
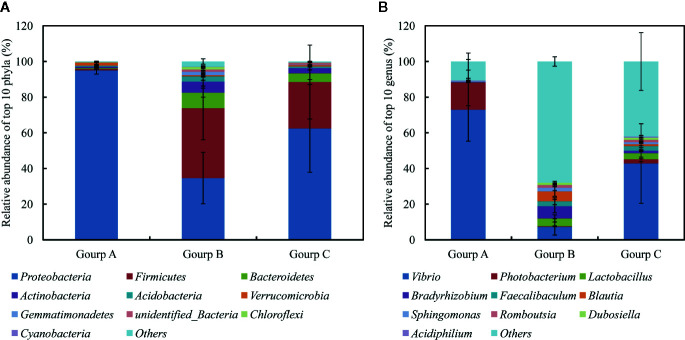
Relative abundances of 10 most abundant phyla **(A)** and genera **(B)**, averaged over each group. Values are means ± SEM (n = 3).

After splicing, quality control, and chimera filtration, a total of 576,940 effective reads were obtained, with an average of 64,104 ± 2,637 reads per sample (mean ± standard deviation) (**Table S2**). The unequal sequencing depths were rarified to 46,764 sequences per sample, resulting in 2,995 operational taxonomic units (OTUs) across all samples. The OTUs were aligned and annotated as 32 known phyla, 52 classes, 122 orders, 233 families, 586 genera, and 329 species. The 10 most abundant phyla were (in decreasing order) Proteobacteria, Firmicutes, Bacteroidetes, Actinobacteria, Acidobacteria, Verrucomicrobia, Gemmatimonadetes, unidentified_Bacteria, Chloroflexi, and Cyanobacteria, which accounted for 64.06, 22.04, 4.65, 3.34, 1.47, 0.98, 0.78, 0.78, 0.57, and 0.17% of the total microbial community, respectively, and together for 94.55–99.95% of the total bacteria (**Figure 5A**). At the genus level, the most abundant phylotypes were affiliated with *Vibrio*, *Photobacterium*, *Lactobacillus*, *Bradyrhizobium*, *Faecalibaculum*, *Blautia*, *Sphingomonas*, *Acidiphilium*, *Dubosiella*, and *Romboutsia*, belonging to Proteobacteria or Firmicutes, which accounted for 48.46, 5.07, 2.80, 2.52, 1.97, 1.89, 1.24, 1.06, 0.95, and 0.30% of the total microbial community, respectively, and together for 28.49–98.00% of the total bacteria (**Figure 5B**).

### Variations in Bacterial Diversity and Compositions Induced by *V. harveyi* Infection

**Figure 6:**
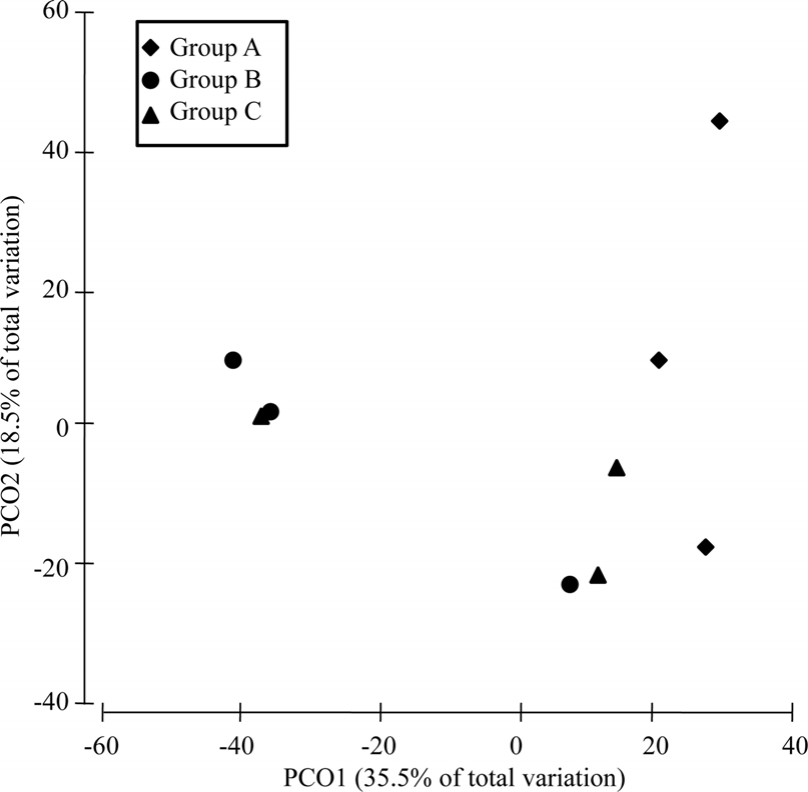
PCoA analysis of the dissimilarity (Bray–Curtis distances) in the intestinal bacterial community compositions of pearl gentian grouper after *V. harveyi* infection.

The α-diversity of the bacterial communities tended to decrease in the order: group B > group C > group A (**Table S2**). PERMANOVA showed that the community compositions differed marginally significantly among the three groups (Pseudo-F = 1.4945, *P*-perm = 0.062). Furthermore, the community dissimilarity of low-virulence-affected group B differed marginally significantly from that of the high-virulence-affected group A (Pseudo-F = 3.2601, P-perm = 0.099), although it was similar to that of the normal-saline-treated control group C (Pseudo-F = 0.9559, P-perm = 0.625). Axis 1 (PCO1) in the PCoA analysis separated high-virulence group A from low-virulence group B and control group C (identified as group B/C) (**Figure 6**). The first two axes (PCO1 and PCO2) explained 35.5% and 18.5% of the variation in composition, respectively (**Figure 6**).

Based on the PCoA results, a SIMPER analysis of the 10 most abundant genera was performed to compare group A and group B/C. The results indicated that *Vibrio*, *Photobacterium*, *Lactobacillus*, *Blautia*, *Bradyrhizobium*, and *Faecalibaculum* accounted for more than 80% (up to 82.01%) of the dissimilarity between the two groups (**Table 1**). The abundances of *Vibrio* and *Photobacterium* were 4.41- and 11.85-fold higher in high-virulence group A than in group B/C, respectively, whereas the abundances of *Bradyrhizobium*, *Lactobacillus*, *Blautia*, and *Faecalibaculum* were 11.56–144-fold lower in group A than in group B/C (**Table 1**).

**Table 1:** Dissimilarities in the total bacterial communities between group A and group B/C determined with a genera-level SIMPER analysis (data are square root transformed).

Genera	Average abundance of group A	Average abundance of group B/C	Average Dissimilarity	Dissimilarity/Standard Deviation	Contribution (%)	Cumulative contribution (%)
*Vibrio*	0.40	0.84	17.40	1.58	32.40	32.40
*Photobacterium*	0.09	0.31	8.35	1.06	15.54	47.94
*Lactobacillus*	0.17	0.02	5.29	1.37	9.86	57.80
*Blautia*	0.14	0.02	4.43	1.08	8.25	66.05
*Bradyrhizobium*	0.17	0.05	4.39	1.05	8.17	74.22
*Faecalibaculum*	0.12	0.01	4.18	1.05	7.79	82.01

### Functional Predictions and Differences Induced by *V. harveyi* Infection

**Figure 7:**
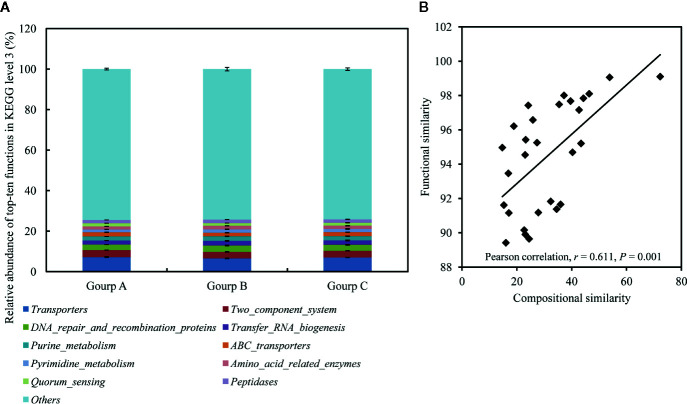
Relative abundances of 10 most abundant functions at KEGG level 3, averaged over each group **(A)**, and the correlations between compositional similarities and functional similarities **(B)**. Values are means ± SEM (n = 3).

In total, 387 KEGG pathways were predicted from the 16S rRNA OTU data with Tax4Fun. The 10 most abundant KEGG pathways at level 3 indicated that the gut microbiota was enriched with pathways associated with transporters, two component system, DNA repair and recombination proteins, transfer RNA biogenesis, purine metabolism, ABC transporters, pyrimidine metabolism, amino-acid-related enzymes, quorum sensing, and peptidases, belonging to the categories *environmental information processing*, *genetic information processing*, *metabolism*, and *cellular processes* (**Figure 7A**). Pearson’s correlation analysis identified a significant positive association between community composition and function (*r* = 0.611, *P* = 0.001; **Figure 7B**).

**Figure 8:**
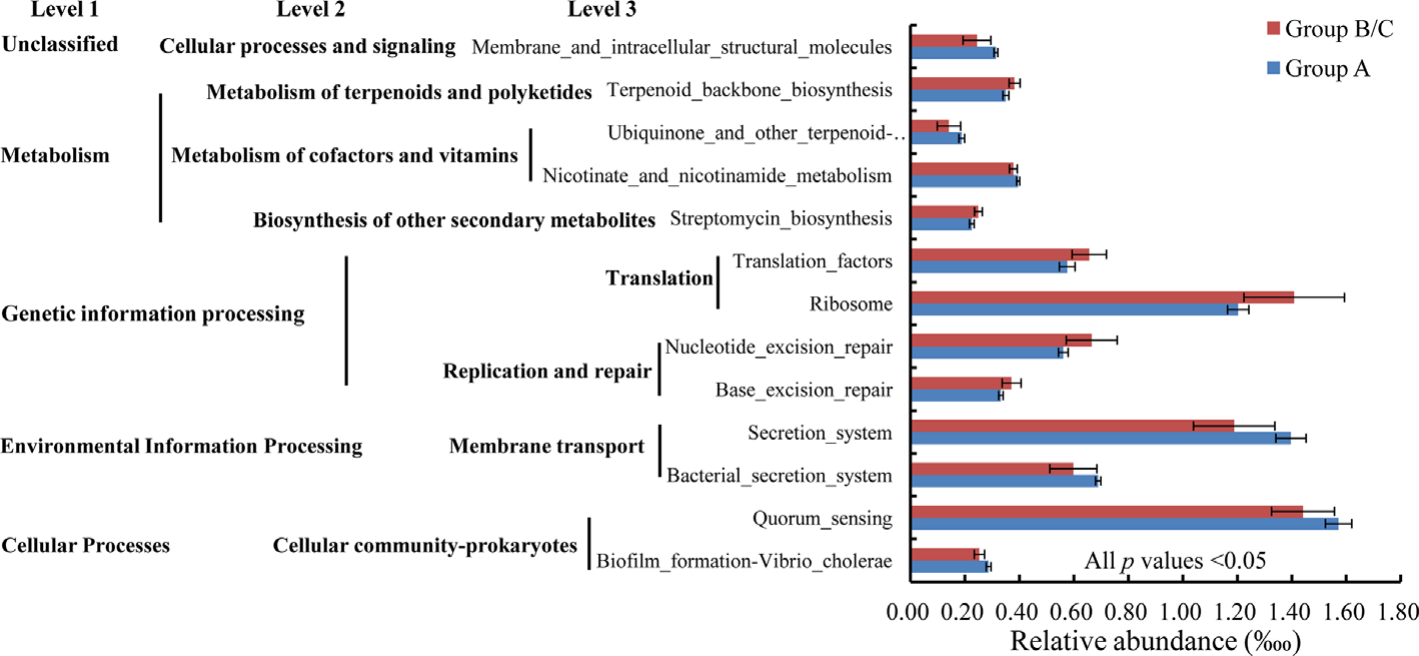
Relative abundances of bacterial functions with relative abundances > 1‱ that differed significantly between group A and group B/C.

A *t* test indicated that 13 functions at KEGG level 3 with relative abundances of more than 1% differed significantly between group A and group B/C. In particular, seven of these functions were induced in high-virulence group A: biofilm formation-*Vibrio cholerae*, quorum sensing, bacterial secretion system, secretion system, nicotinate and nicotinamide metabolism, ubiquinone and other terpenoid-quinone biosyntheses, and membrane and intracellular structural molecules. The other six pathways were reduced in high-virulence group A: base excision repair, nucleotide excision repair, ribosome, translation factors, streptomycin biosynthesis, and terpenoid backbone biosynthesis (**Figure 8**).

### Interactions Within and Among Gut Bacterial Communities, Predicted Functions in the Gut, and the Expression of Immune-Related Genes

Pearson’s correlation coefficients indicated complex interrelationships within and among gut bacterial communities, the predicted functions in the gut, and the expression of immune-related genes (**Table S3**). There were strong negative relationships between *Vibrio* and *Lactobacillus* (r = −0.751, *P* = 0.02), *Blautia* (r = −0.667, *P* = 0.05), or *Faecalibaculum* (r = −0.668, *P* = 0.049), and a positive relationship between *Lactobacillus* and *Faecalibaculum* (r = 0.984, *P* = 0.000). Four of the six indicator taxa (*Vibrio, Lactobacillus*, *Blautia*, and *Faecalibaculum*) were closely associated with the 13 significantly changed functions There tended to be positive correlations between increased taxa and induced functions and between reduced taxa and reduced functions, but negative correlations between increased taxa and reduced functions and between reduced taxa and induced functions (**Table S3**).

The abundance of *Photobacterium* was significantly positively associated with the expression levels of two IFN-induced proteins (r = 0.891 and 0.975, *P* = 0.001 and 0.001, respectively) and one caspases (r = 0.860, *P* = 0.003, respectively). Furthermore, strong associations were comprehensive detected between different predicted functions and between various immune-related genes (**Table S3**).

## Discussion

The pathogenic processes of *Vibrio* include its adhesion, infection, colonization, reproduction, and toxin release, by which it damages the cells and tissues of its hosts, interrupting and destroying the normal metabolism and functions of those hosts (5, 6). After *V. harveyi* challenge, the pathological features, bacterial pathogen load data, and histopathological analyses all indicated that *V. harveyi* colonized the grouper liver, inducing serious pathological changes, including vacuolar degeneration, the accumulation of karyorrhectic debris, pancreatic epithelial cell dissolution, venous blood vessel loss, and connective tissue necrosis. The liver is a central organ of the immune system (17). Several hundred thousand pathogens have been detected in the liver, which suggests that *V. harveyi* may be easily adapt to the liver environment and proliferate (40). The number of high-virulence *V. harveyi* in the liver was much more than that of low-virulence *V. harveyi*, which may be related to the levels of the strains pathogenicity. Compared to the 12 hpi, the increase in the number of pathogens at 24 hpi might be due to the pathogen proliferating in the host, while the decrease in the number of pathogens at 48 hpi might be due to the host immune system attacking the pathogen (40). In addition, no wild-type strain was detected in the liver at 48 hpi probably attribute to the stronger host immune response by injecting high-virulence *V. harveyi*. Here, the colonization of the liver by *V. harveyi* caused an immune response in the liver. In particular, the expression of immune-related genes was altered, including those encoding inflammatory cytokines and receptors (such as IFN-induced proteins), pattern recognition receptors (such as TLR and NOD), and other proteins related to immune cell response (caspase, transferrin, and the transferrin receptor), which are involved in inflammation, iron utilization, pathogen recognition and elimination, *etc.* The immune response caused by the high-virulence strain was stronger than that caused by the low-virulence strain. This is consistent with previous studies (41). IFNs are secreted by host cells, including macrophages, lymphocytes, natural killer cells and fibroblasts, in response to recognition of viral double-stranded RNA intermediates and relating to inflammation (42). Caspases are thought to play a pivotal role in biological phenomena such as cell death and inflammation (43). Transferrin and ferrin are responsible for iron acquisition, transport, and storage and important in the pathogenesis of disease (44). Pattern recognition receptors (TLRs, lectin, and NOD) play essential role in the recognization of specific patterns of microbial components, resulting in antigen elimination and activation of nonspecific inflammatory innate immunity of inflammation (29, 45). Therefore, *V. harveyi* infection attacked the central immune organ, the liver, probably causing an inflammatory response, iron limitation, and cellular apoptosis, resulting in pathological damage to the host and inducing an immune response.

The gut-liver immune system has been well studied in mammals and is gaining tremendous global attention in fish (17). The liver and gut are considered mutually responsive. A damaged gut can expose the liver directly to intestinal endotoxins, and the destruction of the normal liver physiology can cause intestinal dysfunction (46). The fish gut and liver may collaborate immunologically to maintain their homeostasis using strategies specific to both tissues. In this study, although there was no significant change in the intestinal pathology, the immune response in the gut was similar to that in the liver and was markedly induced by *V. harveyi* infection. The intestinal pathology changes during pathogen infection including intestinal mucosal necrosis, atrophy and abscission of intestinal villi, severe edema, congestion and inflammatory cells infiltration, which intuitively indicate the intestinal health (47, 48). For example, tilapia infected with different pathogenic *Streptococcus agalactiae* strains showed different pathology (47). At 3 day postinjection (dpi) infected with low pathogenic *S. agalactiae*, lamina propria were edema, while showed no histopathological changes at 7dpi and 14 dpi. In addition, at 3 dpi infected with high pathogenic *S. agalactiae*, lamina propria showed edema and congestion, and showed severe edema, congestion and inflammatory cells infiltration in lamina propria at 7 and 14 dpi (47). Therefore, the changes of intestinal pathology are related to bacterial virulence, infection time and even infection dosage. In this study, no obvious lesions happened in the intestinal probably attribute to insufficient infection time. However, the GALT, which is connected to the liver by bile and blood, forms a local immune environment for the purposes of both defense and tolerance, so the intestinal immune response is probably mediated by the gut-liver axis (17). The gut microbiota also plays critical roles in epithelial renewal and maturation, which in turn regulate immunological homeostasis, providing essential health benefits to its host. Compared with germ-free zebrafish, zebrafish which is conventionally raised exhibit a greater abundance of genes associated with epithelial proliferation and innate immune response (49). With a commensal microbiota, the germ-free zebrafish can robustly activate NF-kB and its target genes in intestinal and extra-intestinal tissues (50). Additionally, colonization of commensals in larvae stimulates neutrophils and activates pro-inflammatory genes through the TLR/MyD88 signaling pathway and phagocytes, thus enhance disease resistance in zebrafish (51). Additionally, the colonization of probiotics also active innate immune responses and protect fish against pathogens (52). Irianto & Austin (53) demonstrated that the humoral and cellular immunity was stimulated as the lysozyme activity and the number of erythrocytes, macrophages and lymphocytes were increased, after feeding rainbow trout with probiotics containing *Aeromonas hydrophila*, *Vibrio fluvialis*, *Carnobacterium* sp. and *Micrococcus luteus* for 2 weeks. However, pathogens infection is likely to cause intestinal immune stimulation and change intestinal microflora. For example, proinflammatory cytokines IL1β and tumor necrosis factor α (TNF-α), belonging to inflammatory cytokines and receptors, were activated in response to *V. anguillarum* infection in ayu (*Plecoglossus altivelis*), and the relative abundances of 16 taxa in the gut, including Clostridiales, changed consistently during infection (54). Therefore, the changes in the expression of immune-related genes in this study should induce changes in a variety of gut microbiota.

16S rRNA sequencing suggested that the gut microbial α-diversity was lower after infection with high-virulence *V. harveyi* than in the control group and after infection with low-virulence *V. harveyi*. Previous studies have shown that high gut microbial diversity is positively related to host health (55–57) and that the diversity of the gut bacteria decreases significantly during disease progression (58). A PCoA ordination biplot of the 16S rRNA data corroborated the notion that virulent *V. harveyi* infection modulated the total bacterial population of the gut microbiota in the pearl gentian grouper. Similar observations have also been reported for the gut bacteria of the zebrafish, insofar as the taxonomic annotations of the diseased zebrafish clustered together, whereas those of the healthy zebrafish were scattered (59). These findings imply that *V. harveyi* infection drives the gut bacterial community into a kind of diseased state, accompanied by reduced bacterial α-diversity. Highly virulent *V. harveyi* promoted the dysbiosis of the intestinal microorganisms and grouper disease, whereas the gut microbiota of the groupers infected with weakly virulent *V. harveyi* recovered to the state observed in the saline-treated normal control group. Commensal bacteria produce a series of enzymes to digest feed, and nutrients are absorbed into the blood vessels and lymphatic vessels through the intestinal wall and then sent to all the body. In addition, commensals can protect the host by depriving invading pathogens of nutrients, secreting a range of antimicrobial substances and occupying the niche (60, 61). At the species level, no *V. harveyi* was detected in the gut microbiota, confirming that the intestinal immune response was probably caused by the gut-liver axis thus change the gut microbiota.

Our SIMPER analysis indicated that the six most abundant genera were the six most significantly different genera, accounting for more than 80.00% dissimilarity between the two groups defined by their significantly different mortalities (high-mortality group A and low-mortality group B/C). Highly virulent *V. harveyi* infection significantly increased the abundances of predominantly *Vibrio* and *Photobacterium*, whereas the abundances of *Lactobacillus*, *Blautia*, *Bradyrhizobium*, and *Faecalibaculum* decreased significantly. *Vibrio* and *Photobacterium* are well-known opportunistic pathogens associated with various diseases of marine animals, including vibriosis (62) and pasteurellosis (63). An overabundance of *Vibrio* spp. in the gut is reported to parallel disease progression in shrimp and crab (57, 64). *Photobacterium* species are pathogenic in a variety of marine animals, including fish, crustaceans, mollusks, and cetaceans (65, 66). *Lactobacillus*, *Blautia*, *Bradyrhizobium*, and *Faecalibaculum* are all well-known probiotics. *Lactobacillus* species are commensal inhabitants of the gastrointestinal tracts of animals and humans, with anti-pathogenic-bacteria properties and are used for the maintenance of healthy intestinal microflora (67, 68). *Blautia* is known to live in the gut and helps to assimilate nutrients (69). *Blautia hydrogenotrophica* contributes to the breakdown of indigestible components of the host’s diet, predominantly plant materials such as terpenoids and polyketides (8). *Bradyrhizobium* and *Faecalibaculum* are important in nitrogen utilization by the host (70, 71), and in the prevention and treatment of metabolic syndrome, bowel disorders, and certain types of cancer (72–75). Highly virulent *V. harveyi* infection induced changes in these six taxa, indicating an increasing risk of disease in the host. Notably, a strong negative relationship was detected between *Vibrio* and *Lactobacillus*, *Blautia*, or *Faecalibaculum*, and a positive relationship was detected between *Lactobacillus* and *Faecalibaculum*. The abundance of *Photobacterium* was also significantly positively associated with the expression of IFN-induced proteins and caspase. Therefore, gut immunity was induced by the gut-liver axis, causing an imbalance in the interspecies interactions of the gut microbiota. The increase in potential pathogens and the reductions in probiotic bacteria could increase the competition for nutrients and niches in the gut, contributing to the occurrence and progression of disease. Changes in the relative abundances of these indicator bacteria could also be used to predict an increased risk of *V. harveyi* infection in the pearl gentian grouper. According to our preliminary results, the oral administration of live *Lactobacillus* cells in the groupers’ diet for 5 days before their injection with the highly virulent *V. harveyi* strain reduced fish mortality after infection by 10% (**Figure S4**).

The physiological functions of the microbial community were also predicted, to further clarify the damage to the gut microbiota after *V. harveyi* infection. A significant positive correlation between bacterial compositional similarity and functional similarity was observed, indicating relatively low redundancy in the bacterial functions (76). In particular, the KEGG orthology groups induced in the high-mortality group, including biofilm formation–*Vibrio* *cholerae*, quorum sensing, bacterial secretion system, and secretion system, are related to bacterial pathogenesis (77, 78), and this finding was probably attributable to the increased amounts of pathogens present. The reduced metabolism and genetic information processing pathways could weaken the utilization of nutrients and cell functions, thus compromising fish survival and growth. Overall, both the dysbiosis of the intestinal bacterial communities and the disruption of their functions in the pearl gentian grouper caused by the immune changes that occurred during *V. harveyi* infection disturbed the physiological functions of the fish and exacerbated their disease.

In conclusion, this study demonstrated that *V. harveyi* infection induces pathological changes in the pearl gentian grouper. Although no *V. harveyi* was detected in the gut, the gut-liver axis caused parallel immune responses in the liver and gut. This in turn substantially disrupted the composition and functions of the gut bacterial community in the grouper, affecting its survival. Six infection-sensitive gut taxa are potential indicators of *V. harveyi* infection. These findings significantly improve our understanding of the interplay between the gut microbiota and the host immune responses from a microbial ecological perspective, while clarifying the virulence mechanism of *V. harveyi* in the pearl gentian grouper from the perspective of the host’s response. In future work, we will focus on the following two aims: (i) to validate the ecological patterns in a natural infection process that simulates the complex “wild” ecosystem; and (ii) to identify the functions of the four probiotic taxa that were reduced in the group infected with highly virulent *V. harveyi* to establish microbe-based therapies for *V. harveyi* disease in the pearl gentian grouper.

## Data Availability Statement

The datasets presented in this study can be found in online repositories. The names of the repository/repositories and accession number(s) can be found in the article/**Supplementary Material**.

## Ethics Statement

The animal study was reviewed and approved by the Animal Care and Use Committee of the South China Sea Fisheries Research Institute, Chinese Academy of Fishery Sciences, Guangzhou, China.

## Author Contributions

YD conceived the study, analyzed the data, and wrote the manuscript. YZ, HC, and QW performed the experiments. LX critically revised the manuscript. JF contributed the reagents. All authors contributed to the article and approved the submitted version. All authors contributed to the article and approved the submitted version

## Funding

This work was supported by the National Natural Science Foundation of China (NSFC) (31902415), the Natural Science Fund of Guangdong (2018A030310695, 2019A1515011833), Hainan Provincial Natural Science Foundation of China (319QN336), the Central Public-interest Scientific Institution Basal Research Fund, South China Sea Fisheries Research Institute, CAFS (2019TS04), and the Central Public-interest Scientific Institution Basal Research Fund, CAFS (2019ZD0707).

## Conflict of Interest

The authors declare that the research was conducted in the absence of any commercial or financial relationships that could be construed as a potential conflict of interest.

## Supplementary Material

Supplementary Figure 1 Cumulative mortality of pearl gentian grouper after intraperitoneal injection of *V. harveyi* 345:pMMB207 (Group A), *V. harveyi* 345Δ*hfq*:pMMB207 (Group B), and normal saline (Group C).

Supplementary Figure 2 Histopathological changes in different organs of the pearl gentian grouper after infection with *V. harveyi*. Brain **(A)**, heart **(D)**, spleen **(G)**, gill **(J)**, and kidney **(M)** after infection with high-virulence *V. harveyi*. Brain **(B)**, heart **(E)**, spleen **(H)**, gill **(K)**, and kidney **(N)** after infection with low-virulence *V. harveyi*. Brain **(C)**, heart **(F)**, spleen **(I)**, gill **(L)**, and kidney **(O)** after treatment with normal saline. Arrows point to lesions.

Supplementary Figure 3 Expression levels of another 16 (A–P) immune genes in the gut (1, 2, and 3) and liver (4, 5, 6) of the pearl gentian grouper after infection with high-virulence *V. harveyi* (1, 4), low-virulence *V. harveyi* (2, 5), or treatment with normal saline (3, 6). Expression was normalized to the expression in the grouper gut after treatment with normal saline (3) and the ordinate is the normalized expression ratio. One-way ANOVA indicated that no significantly differences were detected in the expression of genes.

Supplementary Figure 4 Cumulative mortality of pearl gentian grouper after intraperitoneal injection of *V. harveyi*. ‘With/without *Lactobacillus*’ means that the fish were or were not administered live *Lactobacillus* cells orally in their diet for 5 days before injection.
