# Supplementary figures and images for: Gut–Liver Immune Response and Gut Microbiota Profiling Reveal the Pathogenic Mechanisms of Vibrio harveyi in Pearl Gentian Grouper (Epinephelus lanceolatus ♂ × E. fuscoguttatus ♀)
Source: Front Immunol. 2020 Nov 26;11:607754. doi: 10.3389/fimmu.2020.607754 (PMC7727329; doi:10.3389/fimmu.2020.607754)

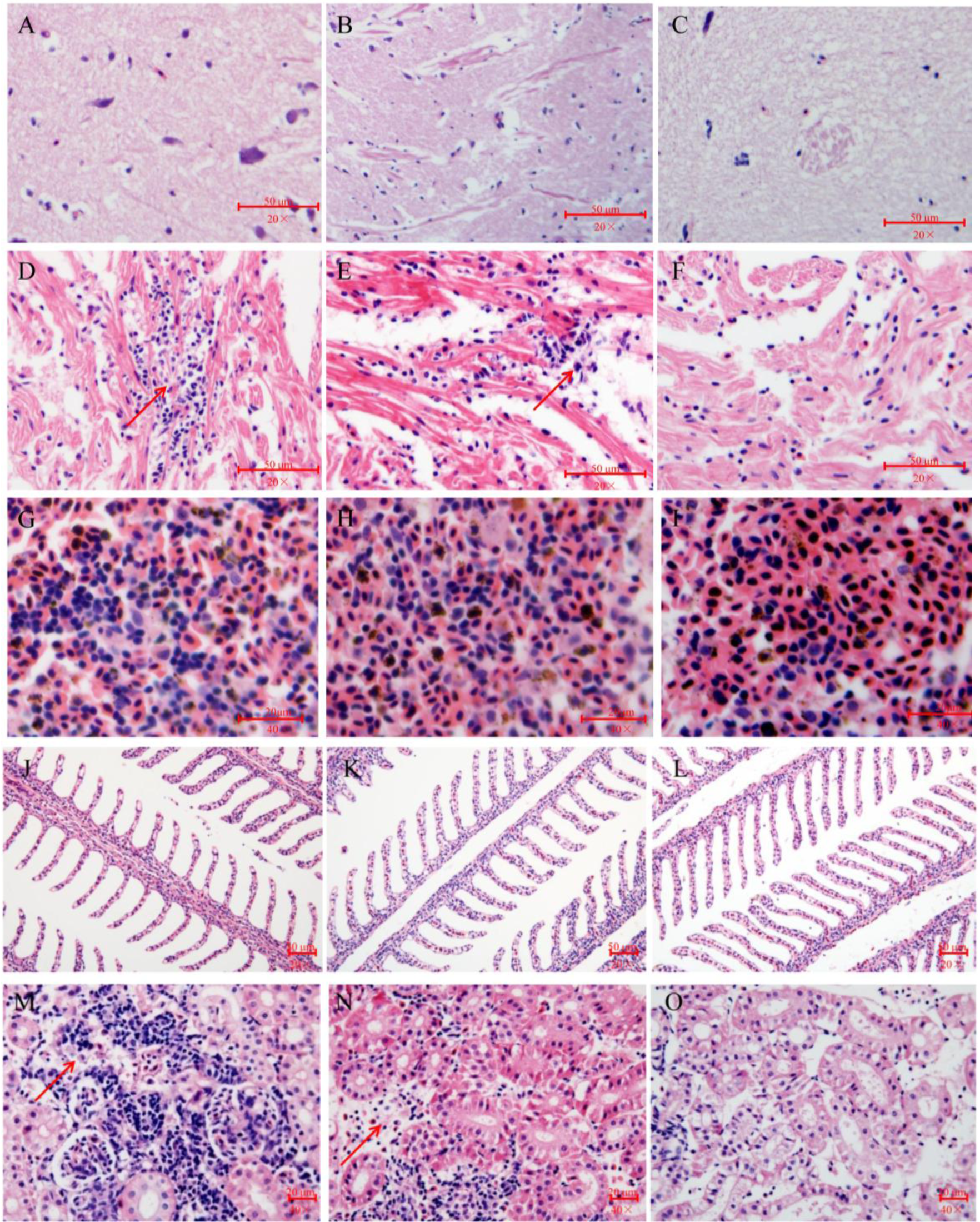

Supplement: Supplementary Figure 2 — Histopathological changes in different organs of the pearl gentian grouper after infection with V. harveyi. Brain (A), heart (D), spleen (G), gill (J), and kidney (M) after infection with high-virulence V. harveyi. Brain (B), heart (E), spleen (H), gill (K), and kidney (N) after infection with low-virulence V. harveyi. Brain (C), heart (F), spleen (I), gill (L), and kidney (O) after treatment with normal saline. Arrows point to lesions. [file Image_2.tif]

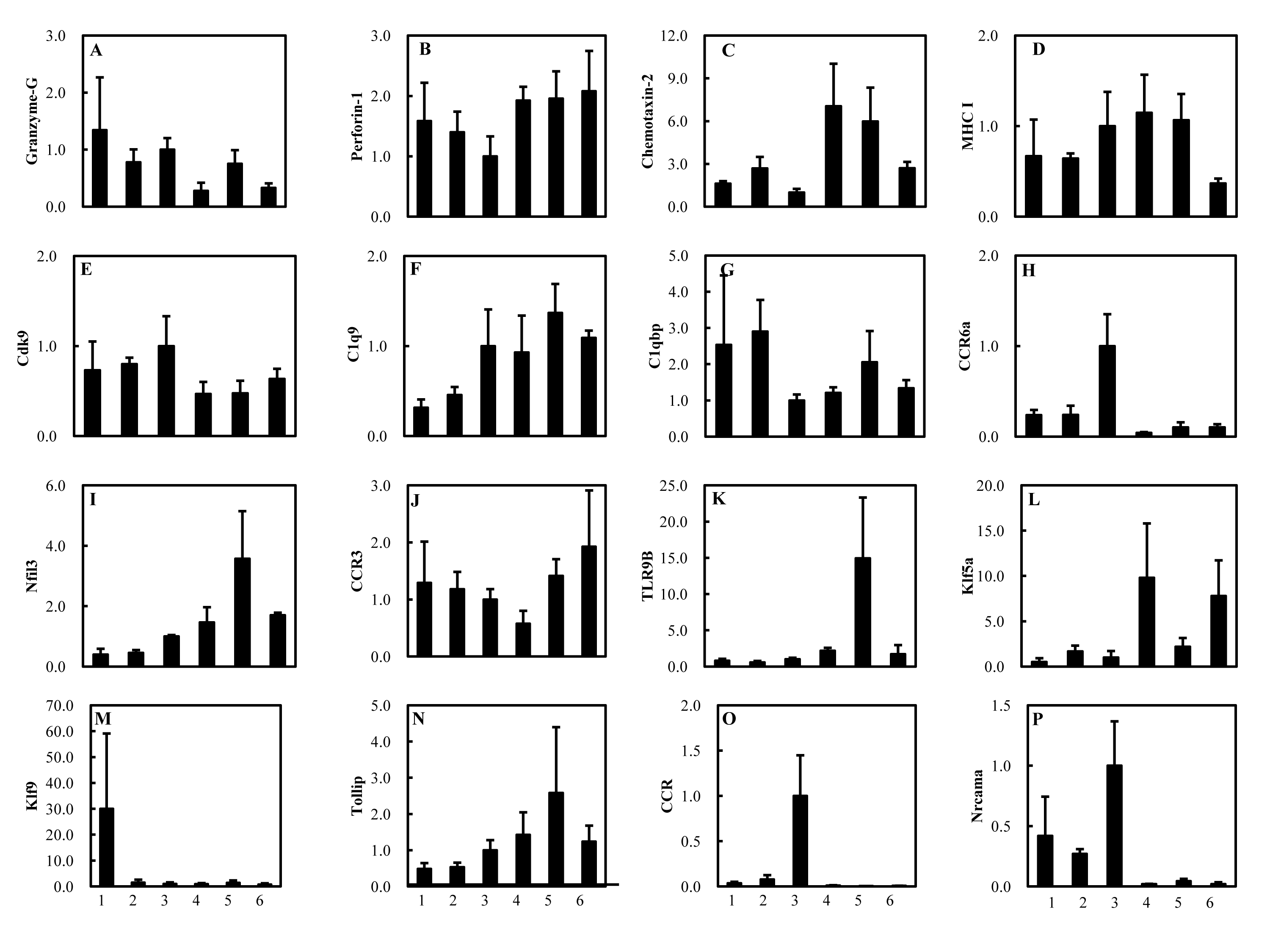

Supplement: Supplementary Figure 3 — Expression levels of another 16 (A–P) immune genes in the gut (1, 2, and 3) and liver (4, 5, 6) of the pearl gentian grouper after infection with high-virulence V. harveyi (1, 4), low-virulence V. harveyi (2, 5), or treatment with normal saline (3, 6). Expression was normalized to the expression in the grouper gut after treatment with normal saline (3) and the ordinate is the normalized expression ratio. One-way ANOVA indicated that no significantly differences were detected in the expression of genes. [file Image_3.tif]
