# Supplementary material for: Gut–Liver Immune Response and Gut Microbiota Profiling Reveal the Pathogenic Mechanisms of Vibrio harveyi in Pearl Gentian Grouper (Epinephelus lanceolatus ♂ × E. fuscoguttatus ♀)
Source: Front Immunol. 2020 Nov 26;11:607754. doi: 10.3389/fimmu.2020.607754 (PMC7727329; doi:10.3389/fimmu.2020.607754)
Supplement: Supplementary file 6 [file Table_2.doc]

**Table S2** Effective taqs and α-diversity.

| Sample name | Effective taqs(#) | observed_species | shannon | simpson | chao1 | ACE | goods_coverage | PD_whole_tree |
| --- | --- | --- | --- | --- | --- | --- | --- | --- |
| Group A | 70046±612^A^ | 780±246^A^ | 2.733±0.646^A^ | 0.667±0.063^A^ | 1043.440±304.743^A^ | 1089.077±297.103^A^ | 0.993±0.002^A^ | 66.310±11.211^A^ |
| Group B | 60338±8346^A^ | 843±45^A^ | 7.028±0.513^B^ | 0.974±0.007^B^ | 973.281±33.759^A^ | 1025.285±31.635^A^ | 0.996±0.000^A^ | 91.048±19.108^A^ |
| Group C | 61929±6479^A^ | 1144±122^A^ | 5.151±0.945^AB^ | 0.844±0.082^AB^ | 1370.698±89.510^A^ | 1414.254±105.294^A^ | 0.992±0.001^A^ | 98.980±14.739^A^ |

Note: The results of One-way ANOVA. Different letters (A, B) represent significant difference, and the same letters represent no significant difference.
